# Evaluation of the SWAN Game‐Based Approach to Re‐Building Numeracy Skills in Aphasia: Feasibility and Preliminary Findings

**DOI:** 10.1111/1460-6984.70256

**Published:** 2026-04-26

**Authors:** Caroline Newton, Vanessa Meitanis, Carolyn Bruce, Chris Donlan

**Affiliations:** ^1^ Division of Psychology & Language Sciences University College London London UK

**Keywords:** acalculia, aphasia, gamification, intervention, numeracy difficulties

## Abstract

**Background:**

Numeracy difficulties are common in individuals with post‐stroke aphasia, yet assessments and therapies addressing these are limited. This study investigated the feasibility and effectiveness of SWAN, a game‐based digital intervention targeting foundational number language skills: counting and transcoding.

**Aims:**

(1) To explore rates of recruitment, retention and adherence to SWAN; (2) To assess whether SWAN improves numeracy skills.

**Methods & Procedures:**

Eighteen individuals with aphasia were given SWAN to play at home for 15 minutes per day over three weeks. Outcome measures included assessments of transcoding, counting, calculation and functional numeracy. Linear mixed‐effects models evaluated intervention effects, with the Smallest Detectable Change (SDC) calculated to identify individuals who responded to the intervention.

**Outcomes & Results:**

SWAN was feasible to deliver remotely, with high adherence. Significant group‐level improvements were observed for transcoding, counting and calculation, although counting was the only outcome where no improvement was observed at baseline. Twelve participants demonstrated meaningful gains in at least one outcome measure, exceeding their individual SDC. Functional numeracy did not improve, though participants reported increased confidence in skills.

**Conclusions & Implications:**

The findings suggest that SWAN is effective in motivating users and providing intensive practice for number sequences. However, further research is required which explores individual response to intervention in order to determine those most likely to benefit.

**WHAT THIS PAPER ADDS:**

*What is already known on this subject*

- Numeracy deficits are prevalent in aphasia, impacting daily living and independence. Limited previous research involving tailored intervention delivered individually suggests these difficulties may be responsive to intervention. However, intervention to address them clinically appears rare.

*What this paper adds to the existing knowledge*

- This study demonstrates the feasibility and efficacy of a game‐based intervention to improve foundational numeracy skills in aphasia, including counting, transcoding and simple arithmetic (addition and subtraction). However, further exploration of SWAN's effectiveness at individual level is required to determine those most likely to benefit from the intervention.

*What are the potential or actual clinical implications of this work?*

- The findings highlight the importance of considering numeracy in aphasia assessment and intervention. Digital therapies like SWAN can offer accessible and engaging solutions for addressing this largely unmet need.

## 1 Introduction

The ability to work with numbers is essential for everyday life. Whereas for most people the frequency with which we encounter numerical information goes largely unnoticed, those for whom this is an area of difficulty are likely to experience a marked impact on many activities (e.g. exchanging phone numbers; identifying the best shopping deal). Such difficulties may be hard to circumvent: the unique precision of number word meanings often makes substitution of one number with another of similar magnitude semantically implausible. There have been several recent reports that a high proportion of the general working population have poor numeracy abilities, impacting financial decisions and health outcomes (e.g. Rowlands et al. 2015). One group where difficulties in number processing and calculation have been reported is individuals with post‐stroke aphasia (see, for example, Seron 2001).

### 1.1 Numeracy Difficulties in Aphasia

Although some people experience spontaneous recovery post‐stroke (Basso et al. 2005), and others retain mathematical skills (Varley et al. 2005), there is now a wide body of research which indicates that a high proportion of people with aphasia may have substantial numeracy difficulties, with poorer performance than non‐aphasic people on a range of skills. This includes the count sequence (e.g. De Luccia and Ortiz 2014; Marangolo et al. 2005) and transcoding—that is, converting numbers between Arabic numerals and their spoken and written names (e.g. Marangolo et al. 2004; Messina et al. 2009). Deficits are also observed on basic numerical comprehension tasks which do not require language production (e.g. Proios et al. 2021), and in calculation, with deficits often greatest in multiplication (e.g. De Luccia and Ortiz 2014). Detailed analyses in single case studies reveal islands of strength and difficulty within individuals as well as dissociations between number language and broader language abilities.

Difficulties extend to more functional measures of numerical ability, which evaluate skills within the context of daily life activities, for example, shopping, cooking (Ichikowitz et al. 2023; Proios et al. 2021), often resulting in a significant emotional and practical effect. For example, the individuals interviewed by Benn and colleagues (2022) spoke of the impact of such difficulties, particularly among those for whom maths was central to their life before their stroke. Despite developing their own strategies, participants reported relying on others and that this impacted their independence and confidence.

### 1.2 Interventions for Numeracy Difficulties

Despite the significance of numeracy difficulties, intervention to address them clinically appears rare (Benn et al. 2022) and while there are several research studies exploring the rehabilitation of numerical skills in this population, these often focus on calculation, most frequently the treatment of problems with multiplication (e.g. Zaunmuller et al. 2009). Fewer studies have addressed counting and transcoding, though the latter was the focus of all four intervention case studies published to date (Deloche et al. 1989; Deloche et al. 1992; Ablinger et al. 2006; Lemanissier et al. 2023). All included extensive “drilling” practice, with structured progression through stages of increasing difficulty.

For example, significant improvements in transcoding were observed for GG who took part in Ablinger et al.'s (2006) study. She was only able to read aloud Arabic numerals with the assistance of an “automatic counting strategy” (i.e. counting up from 1). The therapy required GG to read aloud Arabic numerals without making use of the strategy. The complexity of the numbers trained (single digits to teen numbers to decades) was gradually increased, and a cueing hierarchy gradually decreased in later stages of the therapy. Significant improvements after therapy were maintained six months later indicating stable effects of the intervention.

The most recent study (Lemanissier et al. 2023) aimed at more controlled evaluation of the effectiveness of intervention by including multiple baseline measures as well as measurements at several points during and after the intervention. The intervention was personalized to the two participants, one of whom had a relatively mild impairment of transcoding involving the spoken number name, the other with a more severe deficit across all transcoding modalities. The therapy involved a complex series of steps, ranging from silent reading to copying the number in the same modality to completing the transcoding. Both women made significant improvements in the transcoding modalities which proved most problematic pre‐therapy, with gains maintained after one month.

The success of these interventions suggests that this is a promising area to address. Numerical skills may be particularly well‐suited to digital intervention which can provide the intensive practice required whilst avoiding costly therapist time and long intervention sessions which are fatiguing for individuals (Lemanissier et al. 2023). There are several commercially‐available apps which include components aimed at improving numerical processing and calculation, for example, Tactus Number Therapy (Tactus Therapy Solutions; Vancouver, Canada) and React2 (React2 Ltd; Peebles, Scotland). Only one app has been the subject of published research exploring its effectiveness.

Constant Therapy (Constant Therapy Health; Lexington, MA) provides an extensive collection of evidence‐based exercises for a range of linguistic and cognitive difficulties experienced by people with aphasia, including maths. While efficacy studies show positive effects of the training on linguistic skills, they provide limited evidence of success with respect to improvements in domains relating to numeracy. Improvements were reported on the training tasks undertaken (Des Roches et al. 2015), but not on outcome measures that included calculation (Braley et al. 2021). This suggests that numerical and calculation skills can be trained via app‐based intervention, but it is unclear whether gains can be generalised to non‐therapy tasks.

### 1.3 The Current Study

The current study evaluates the feasibility of a novel game‐based digital therapy known as SWAN (“Sequences in Words and Numbers”) for ameliorating a range of difficulties in number processing and simple calculation in post‐stroke aphasia. The intervention, completed at home on a hand‐held tablet, focuses on the foundational number language skills of transcoding and counting. It incorporates three key components:

#### 1.3.1 Gamification

The target behaviours (number sequence identification and association of spoken and Arabic forms) operate as core activities within a computer game, rather than a decontextualised training programme. Gamification has been defined as “the use of game design elements in non‐game contexts” (Deterding et al. 2011, p. 9). Relevant elements incorporated into SWAN include game interface design patterns such as points for completing tasks; game mechanics such as tile matching and sequence building and game design principles such as rule‐based, goal‐oriented play, progressive difficulty and immediate feedback on user actions. Gamification has been employed in numeracy interventions aimed at children (e.g. Outhwaite et al. 2019), but to our knowledge, no comparable interventions exist for adults. Engaging the user in a gaming activity which is intrinsically motivating facilitates repeated practice of target behaviours over several sessions spaced out over time. Numerous studies have shown that verbal (re‐)learning is greater following practice that is distributed across multiple sessions rather than completed in a single session (e.g. Hickin et al. 2023), and indeed this was a key principle in the numeracy interventions described above.

#### 1.3.2 Developmental Progression

The number sequences on which the game is based increase in range and complexity in a way which corresponds to the pattern observed in children's learning (Fuson 1988). Previous intervention studies have used progressive levels of lexical and syntactic complexity of numbers in order to address numerical difficulties in aphasia (Ablinger et al. 2006; Deloche et al. 1992). Our approach differs by emphasizing the re‐learning of foundational number language skills. This is grounded in the observation that certain phenomena typically seen in the early stages of numerical development are also evident in adults with aphasia. For example, the individuals who, like GG mentioned above, are unable to enter the number sequence at points beyond 1. We propose that some of these aphasic difficulties may reflect a regression to an earlier developmental stage, a pattern also noted in agrammatism (Dyson et al. 2022). Based on this, we hypothesised that an intervention that guides individuals through developmental stages of numerical learning may facilitate significant improvements.

#### 1.3.3 Hebbian Learning

The design of the game is based on established principles of learning which have particular application to the Arabic number sequence and the spoken number words to which it corresponds. The general principle of correlational or Hebbian learning (Hebb 1949) is applied through repeated selection of consecutive elements of the number sequence. Each correct selection serves to strengthen the association between elements, and provides concurrent linkage between written and spoken forms. Within each matrix of numbers presented, the target (consecutive) number is always included within the limited set of adjacent items, thereby reducing both perceptual and conceptual demands on the learner, as well as the potential for error. Each correctly selected number operates as a therapeutic element which may serve to strengthen existing associations or to rebuild associations which have become degraded.

The study aims to determine the feasibility and acceptability of the SWAN game‐based intervention and to explore its effects on numeracy skills. The research questions examined were:

1. What are rates of recruitment, retention and adherence to SWAN?
2. Is SWAN acceptable to participants?
3. Does SWAN result in improvements to foundational number language skills (counting and transcoding)?
4. Does SWAN impact distal abilities such as simple arithmetic and functional skills?

We hypothesised that SWAN would be most effective for difficulties in proximal skills (i.e. skills which closely match behaviours entailed in playing the game), but also that distal abilities might be improved. This hypothesis was based on evidence from research with children which shows that transcoding and number sequence knowledge are important predictors of later arithmetic ability (e.g. Habermann et al. 2020): here we hypothesised that re‐learning of the former might aide improvements in the latter. Previous studies in aphasia are also encouraging in this respect as they suggest positive changes on undrilled numerical skills may be observed after therapy (e.g. Deloche et al. 1989).

## 2 Methods

### 2.1 The Context of the Study

The study took place between April and August 2021 while Covid‐19 pandemic restrictions were in place across the UK. Consequently, all screening, testing and training on the SWAN app was conducted online using the online experiment building platform Gorilla (https://gorilla.sc/) and Zoom (https://zoom.us/), implementing the screen sharing function to administer assessments. Testing and training were conducted by the second author and three research interns who received six hours of training on assessment and technical procedures and who were provided with a detailed protocol for each testing session. Ethical approval was granted by the university ethics committee.

### 2.2 Study Design

**FIGURE 1:**
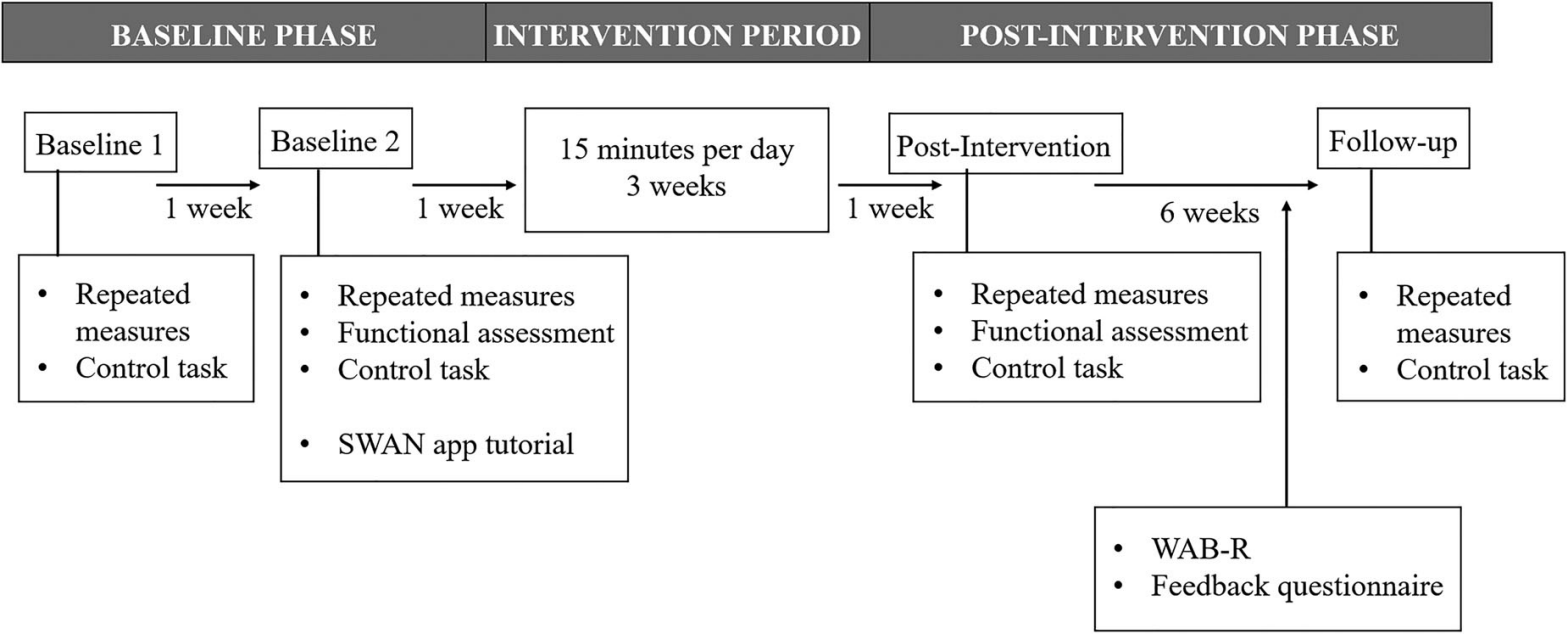
Study design.

A within‐subjects design was used, with outcome measures administered twice at baseline (one week apart), post‐intervention and at a six week follow‐up (Figure 1). During an initial session, participants provided formal consent and completed a task to practise screen‐sharing and computer mouse control.

### 2.3 Participants

**FIGURE 2:**
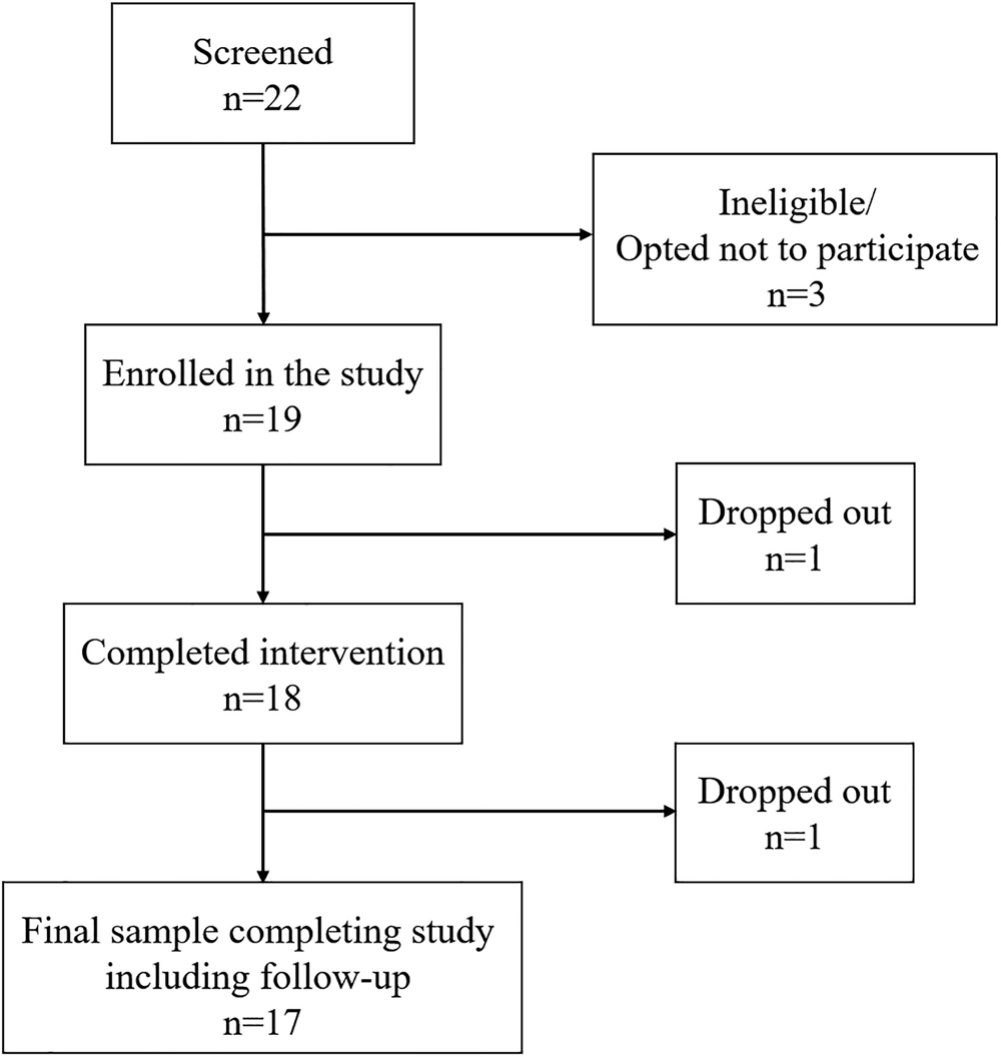
Flow chart of participant recruitment.

Participants were recruited via social media and word of mouth, and a university communication clinic research register. Recruitment targeted adults with aphasia experiencing difficulties recognizing, reading or writing numbers or with counting. All those who expressed interest in the study were screened for their eligibility. Screening consisted of brief assessment of transcoding and counting skills and a conversation about the everyday impact of their numerical difficulties (Figure 2). Individuals were excluded from the study if they performed without error on the screening tasks and/or had any physical difficulties that would limit their ability to use the SWAN application on a tablet device.

Information on the participants who completed the intervention study is shown in Table 1, which includes details of the type and severity of their aphasic difficulties, as identified using the Western Aphasia Battery—Revised (WAB‐R, Kertesz 2007).^1^ Four participants had severe or very severe Broca's aphasia, four had Conduction aphasia and 10 had anomic aphasia; there were no individuals with Wernicke's aphasia. The group included four women and 14 men, aged 37–85 years.

**TABLE 1:** Participant demographic information.

					Western aphasia battery	
Participant	Age	Gender	Highest education level	Current/previous employment	Aphasia quotient	Aphasia type
A20	47	F	College/university	Teacher	92.9	Anomic
A22	70	M	College/university	Engineer	24.3	Broca's
A23	48	F	Secondary school	Finance officer	96.1	Anomic
A24	63	M	College/university	Engineer	78.3	Anomic
A25	85	M	College/university	Judge	84.4	Conduction
A27	77	F	College/university	Civil servant	71.1	Conduction
A29	71	F	Postgraduate	Teacher	35.9	Broca's
A30	72	M	College/university	Accountant	92.6	Anomic
A31	76	M	College/university	Computer analyst	69.7	Conduction
A32	37	M	Postgraduate	Managing director	78.8	Anomic
A33	61	M	College/university	Upholsterer	84.4	Anomic
A34	71	M	Postgraduate	Solicitor	78.7	Anomic
A35	64	M	College/university	Civil servant	45	Broca's
A37	53	M	Postgraduate	Photojournalist	96.4	Anomic
A38	71	M	Postgraduate	Teacher	61.5	Conduction
A39	39	M	College/university	Recruitment	93.2	Anomic
A40	57	M	Postgraduate	Programmer	46.9	Broca's
A41	63	F	College/university	Nurse	85.5	Anomic

### 2.4 Outcome Measures

Measurements aimed to detect growth in proximal and distal skills. Assessments of proximal goals comprised two tests of foundational number language: transcoding (including number identification, number reading and number writing) and rote counting (including forwards and backwards counting). Distal goals (i.e. skills more distantly related to SWAN gameplay) comprised calculation (including addition and subtraction) and functional numeracy skills. Lists of items included in unpublished numerical tasks are included in Appendix A.

#### 2.4.1 Transcoding

This assessment included writing numbers to dictation, identification of spoken numbers and reading Arabic numerals aloud. Targets included single, double and triple digits, selected to elicit phonological, semantic or syntactic errors reported in the aphasia literature (e.g. Messina et al. 2009). Targets were identical across all timepoints, but presented in different orders.

##### 2.4.1.1 Number Writing

The tester read aloud a set of spoken number names which participants transcribed as Arabic numerals on paper. After every five items, the participant held up the paper to the camera for scoring.

##### 2.4.1.2 Number Identification

Participants were presented with four Arabic numerals and were asked to select the numeral which matched a spoken number name. For this sub‐task, the tester shared their screen and gave the participant control of the mouse.

##### 2.4.1.3 Number Reading

Participants read aloud Arabic numerals presented on the screen, while testers recorded whether responses were correct or incorrect.

One point was awarded for each correct answer and the maximum score was 60. The Cronbach's alpha reliability for the test using scores at Baseline 1 was 0.96.

#### 2.4.2 Counting

Participants were asked to count aloud forward or backward from a given number for eight sequences of five numbers in each direction (e.g. 17–18–19–20–21). Sequences included allowed us to assess participants’ ability in single, double and triple digits and to cross critical boundaries (decades and hundreds).

The scoring system was based on single items so that one point was awarded for each number in the count sequence produced, with a maximum score of five for each sequence, and of 80 for the task. This procedure differs from tasks used elsewhere (e.g. Delazer et al. 2003) where a single point is awarded for an entire sequence correctly produced. However scoring based on single items provided greater sensitivity for crediting partially completed sequences. Full details of count scoring procedures are provided in Appendix B. The Cronbach's alpha reliability for the test at Baseline 1 was 0.95.

#### 2.4.3 Calculation

The assessment included simple addition and subtraction problems similar to the Test of Basic Arithmetic and Numeracy Skills (TOBANS; Brigstocke et al. 2016). Use of a timed task allowed us to evaluate the efficiency and fluency of participants’ skills, for example, use of counting strategies versus retrieving answers from memory, which untimed tests would not have the sensitivity to detect. The four sub‐tasks each included 60 items:

##### 2.4.3.1 Addition

Problems where the addends were single digit numerals and where the sum was less than 10 (e.g. 2 + 5).

##### 2.4.3.2 Addition With Carry

Problems with single digit addends requiring a carry operation where the sum was between 11 and 18 (e.g. 9 + 7).

##### 2.4.3.3 Subtraction

Problems with single digits where the solution was between 1 and 7 (e.g. 7–2).

##### 2.4.3.4 Subtraction With Carry

Problems where the subtrahend was a single digit and the minuend was a number in the teens, with the solution between 2 and 9 (e.g. 13–5).

The tester gave the participant control of the screen in order to complete this task. All problems were presented in Arabic notation and participants typed their answer on screen using Arabic numerals. For each sub‐task, participants were given 60 seconds to solve as many problems as possible. Problems were presented in a set order across participants and timepoints. One point was given for each correct answer (maximum: 240), so that the total score reflected the number of problems correctly solved within the time limit.

#### 2.4.4 Functional Numeracy

The 23‐item Functional Numeracy Assessment (FNA, Ichikowitz et al. 2023) was used. Because of the length of the test and the overall volume of online testing, functional numeracy was tested twice: once before and after intervention. Pearson correlation analyses revealed significant positive correlations between the FNA and the assessments of transcoding (*r*(18) = 0.68, *p* = 0.002), counting (*r*(18) = 0.63, *p* = 0.005) and calculation (*r*(18) = 0.84, *p* < 0.001).

#### 2.4.5 Control Measure

**FIGURE 3:**
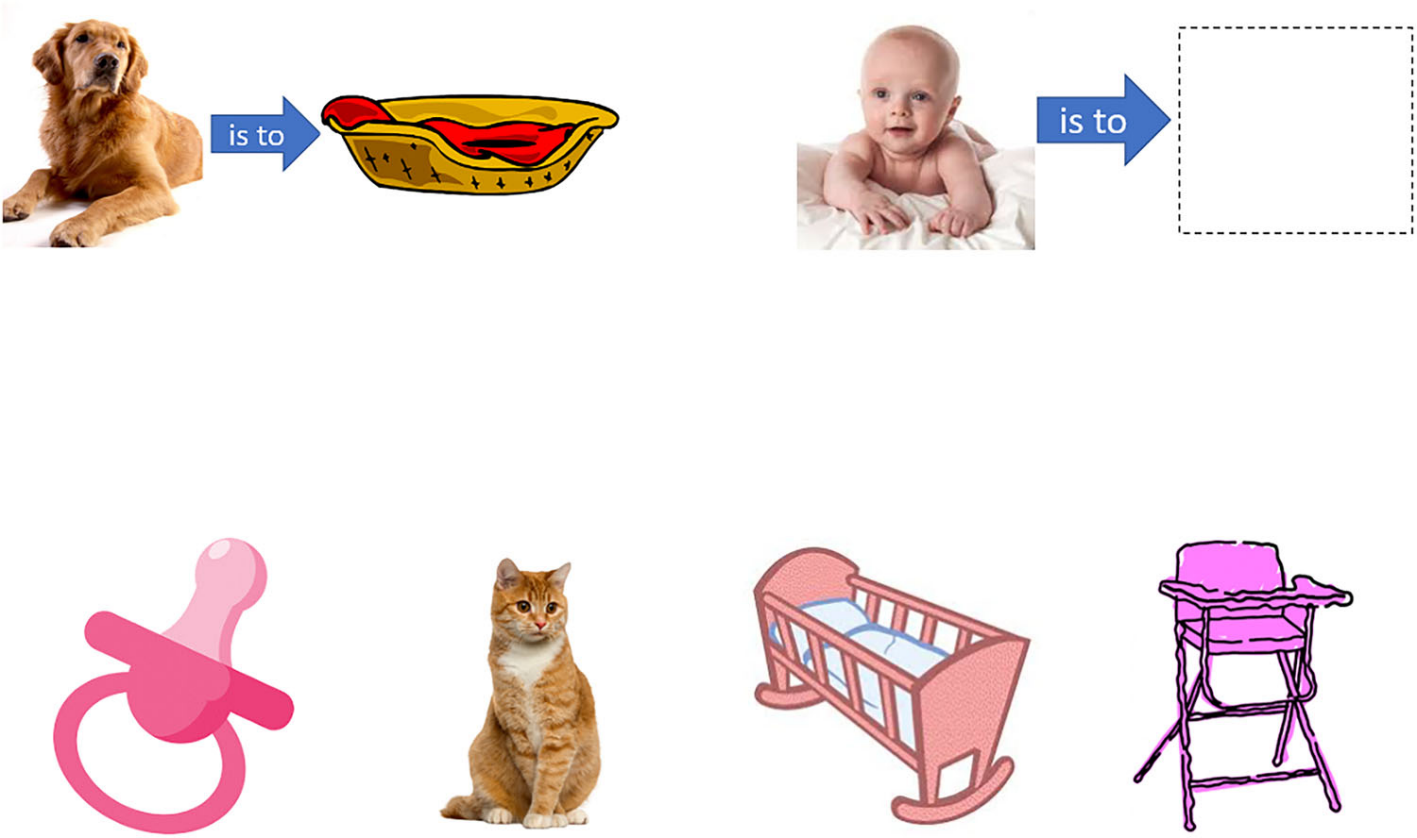
Item from the picture analogy control task.

The assessment battery also included an unpublished non‐numerical control task, based on analogical reasoning. Performance was expected to remain stable, thereby providing an indication of the specificity of any post‐intervention change in numerical skills. The test uses an “A:B as C:D” forced‐choice paradigm: participants are shown images A, B, and C and are given a choice of four images to complete the relationship with image C (see Figure 3, where the answer is the crib). The test includes 20 items with one demonstration and one practice item. Relationships tested include antonymy (dirty: clean as happy: sad), cause and effect (freezing conditions:frostbite as bright sunlight: sunburn) and part to whole (nose: face as foot: leg). The participant was given control over the mouse to register their responses. One point was awarded for each correct answer. The Cronbach's alpha reliability for this test at Baseline 1 was 0.78.

#### 2.4.6 Qualitative Feedback

Participants’ views on SWAN were collected post‐intervention using an anonymous aphasia‐friendly questionnaire which explored their perceptions of SWAN including any changes they would make, the length and intensity of the intervention and whether they observed any benefits.

### 2.5 Intervention

During the intervention period, participants were asked to complete 15 min of SWAN playing time every weekday for three weeks, totalling 3 h 45 min of treatment. Where participants did not have their own Android tablet, a Samsung Galaxy Tab A8 was sent to them. Training on the SWAN app was provided at the second baseline, which ensured they could access the game and that they understood the principles of the game. No randomisation or blinding was used.

**FIGURE 4:**
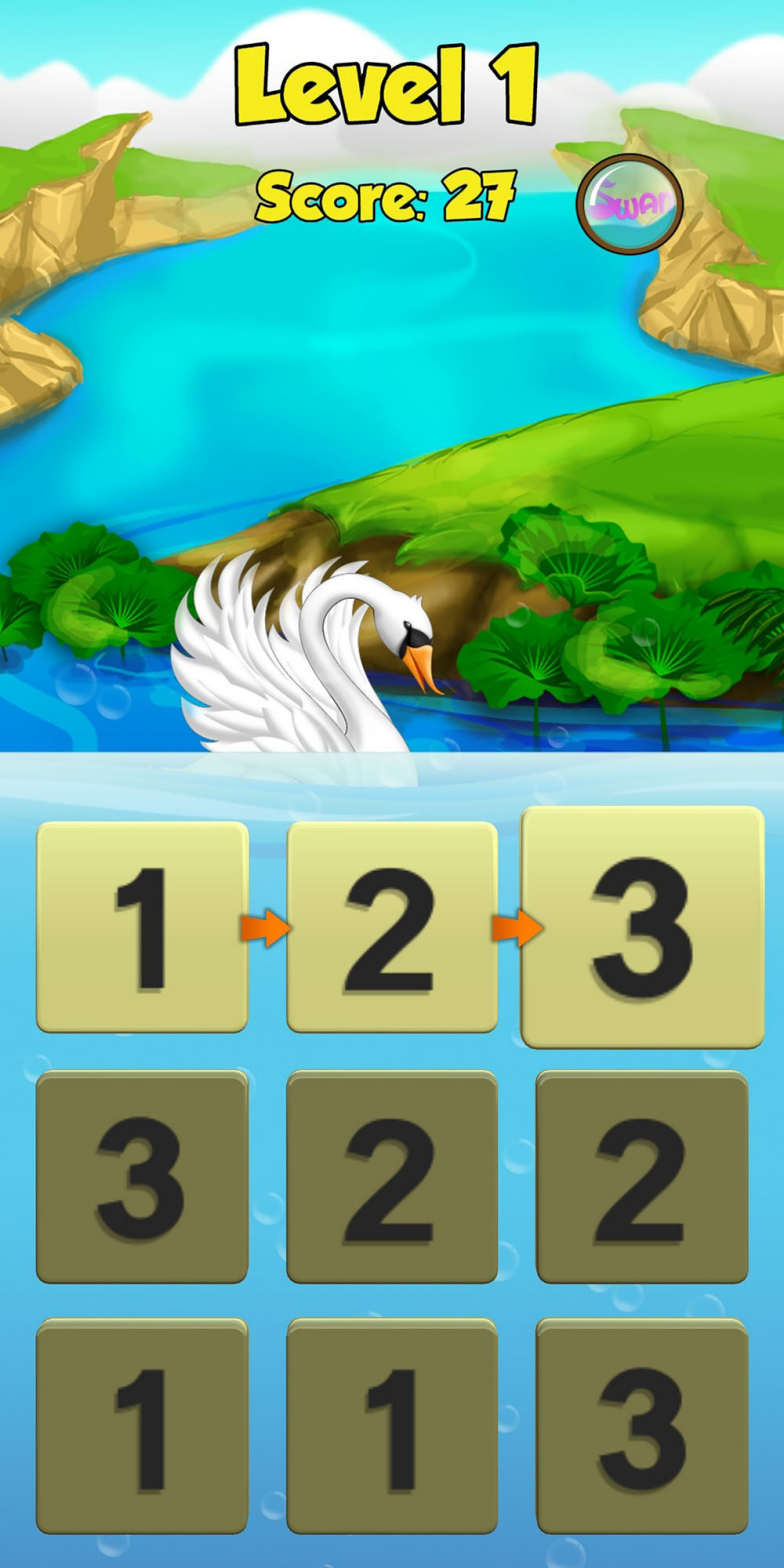
Example screenshot from SWAN Level 1.

The SWAN therapy follows progression through 140 levels. The core activity is the identification the longest possible number sequence available by selecting adjacent tiles in consecutive order (see Figure 4 for an example “game board” from Level 1). Scores are awarded based on the length of each sequence relative to the maximum possible sequence. Every time the player touches an Arabic numeral on the screen they hear the corresponding spoken form. In order to provide support for players with limited knowledge, where a player is slow to start ‘help’ arrows appear on screen indicating a possible sequence. Access to successive levels is determined by the player's score: 70% of the maximum possible score is required, with the score representing a composite based on the number, length and complexity of number sequences identified by the player. Where their score is not sufficient, they are required to repeat the level. A full description of the intervention using the Template for Intervention Description and Replication (TIDieR) checklist is in Appendix C.

#### 2.5.1 Gameplay Data

Every press of every tile, its on‐screen location and timing in relation to other actions, is captured by the game. Particular importance was attached to: sequence length (relative to the longest possible sequence available); a count of errors made (i.e. tiles pressed which did not satisfy the requirements of adjacency or consecutive order) and maximum level reached. Each of the measures was recorded for each sequence entered and summarized for each level. In addition to key SWAN gameplay variables, we followed Cordella and Kiran (2024) by calculating key components related to the intensity of the intervention. Full descriptions of all key gameplay variables are provided in Appendix D. Data capture also allowed observation of a player's adherence to the intervention as a measure of treatment fidelity, as well as the frequency and intensity of each individual's engagement with SWAN (see Appendix D for a heat map showing distribution of gameplay).

### 2.6 Statistical Analysis

Linear mixed effects models were used to examine the effect of SWAN on outcome measures using the lme4 package in R 3.5.1 (Bates et al. 2023). Time was treated as a fixed effect, with Participants included as a random effect factor. Each model was fit using Restricted Maximum Likelihood (REML) and evaluated with Satterthwaite's method. Random intercepts from each analysis were high indicating considerable variability between individuals. Therefore, in addition to group analyses, for each measure which was assessed twice at baseline we identified individuals’ responsiveness to the intervention by calculating the Smallest Detectable Change (SDC) for each person, using their mean score and standard deviation, across the two baselines. The SDC represents the smallest change in a score which could be interpreted as real change in performance rather than measurement error with 95% confidence, and is calculated using the formula: Standard Error of Measurement (SEM)×1.96×√2​ (Breitenstein et al. 2023). Participants were identified as responders if they achieved a change in score from their best performance at baseline to post‐intervention greater than their individual SDC. The SDC accounts for measurement error and baseline variability, therefore participants with lower baseline scores and lower SDC thresholds may register modest absolute changes which qualify as meaningful responses. Conversely, larger absolute changes may not exceed SDC for participants with higher measurement variability. As functional numeracy was assessed twice, any improvements post‐intervention were evaluated with a t‐test for dependent samples using the package “tidyverse” (Wickham et al. 2019).

#### 2.6.1 Missing Data

One participant (A35) was not able to complete the full study as he experienced a stroke before the follow‐up assessment. He was therefore excluded from group analyses, but included in individual SDC calculations.

## 3 Results

### 3.1 Number Processing Profiles

Baseline performance on number processing tasks of transcoding and counting shows variability within individuals across the two testing points, as well as between participants (see Table 2). Some participants (A23, A27, A30, A39) demonstrated largely high performance across tasks; two participants (A22, A40) with Broca's aphasia showed a severe impairment. Other participants exhibited a more complex set of profiles with areas of relative strength and difficulty, including a group with a milder but still pervasive deficit across tasks (A31, A32, A35, A38), as well as individuals with strengths in transcoding but poor counting (A34, A37, A41). Performance patterns on the transcoding subtasks further highlight modality effects (see Appendix E). Three participants (A20, A24, A25) achieved high scores in identification and reading but struggled markedly with number writing. A29, A31 and A38 displayed a more severe impairment on output tasks (writing and counting), whereas A33 showed the reverse pattern, performing weakest on the identification task that taps comprehension.

**TABLE 2:** Pre‐intervention number processing and calculation skills (mean scores at baseline) and individual response to intervention.

Pt	Pre‐intervention mean score			Post‐intervention Increase greater than SDC?			
	Transcoding (max = 60)	Counting (max = 80)	Calculation	Transcoding	Counting	Calculation	Control
A20	52.5	59	54.5		✓		✓
A22	7.5	0	28.5				
A23	60	71.5	41				✓
A24	47.5	46	45		✓	✓	✓
A25	54	63	59				
A27	53.5	68	33	✓	✓	✓	
A29	27	0	9	✓			
A30	58	67.5	55.5		✓	✓	
A31	28.5	13	42		✓	✓	
A32	16	19.5	0	✓	✓		
A33	52	50	36.5			✓	
A34	56	54	50	✓		✓	
A35^a^	29.5	13.5	25	✓			✓
A37	56	59	48.5	✓	✓	✓	
A38	32.5	24	41	✓		✓	
A39	57	71	58				
A40	12.5	1	19.5	✓		✓	
A41	52.5	38	43.5	✓	✓		

^a^ A35 did not complete the follow‐up assessment.

We also examined errors in the writing task to explore whether participants presented primarily with semantic or syntactic deficits (Appendix E). Across the group, there was a predominance of semantic errors (e.g. “fourteen” → 18). In contrast, syntactic errors involving expansion (e.g. “one hundred and three” → 1003) or inversion (e.g. “five hundred and eight” → 580) were rare, with only A41 showing a majority of syntactic errors. Phonological errors (confusions between phonologically similar number words, e.g. “fourteen” → 40) occurred in seven participants but typically co‐occurred with semantic errors rather than appearing in isolation.

There was an association between number processing and individuals’ broader aphasic difficulties: significant high correlations were found between the number reading section of the Transcoding task and the WAB object naming test (both spoken output tasks): *r*_s_(15) = 0.72, *p*<0.01, and between number identification and the WAB auditory word recognition (both auditory input processing tasks): *r*_s_(15) = 0.84, *p*<0.01. However, individual difficulties were not entirely attributable to broader language problems; for example, A32 exhibited severe impairments in numerical tasks despite relatively mild language difficulties.

### 3.2 Response to Intervention

**FIGURE 5:**
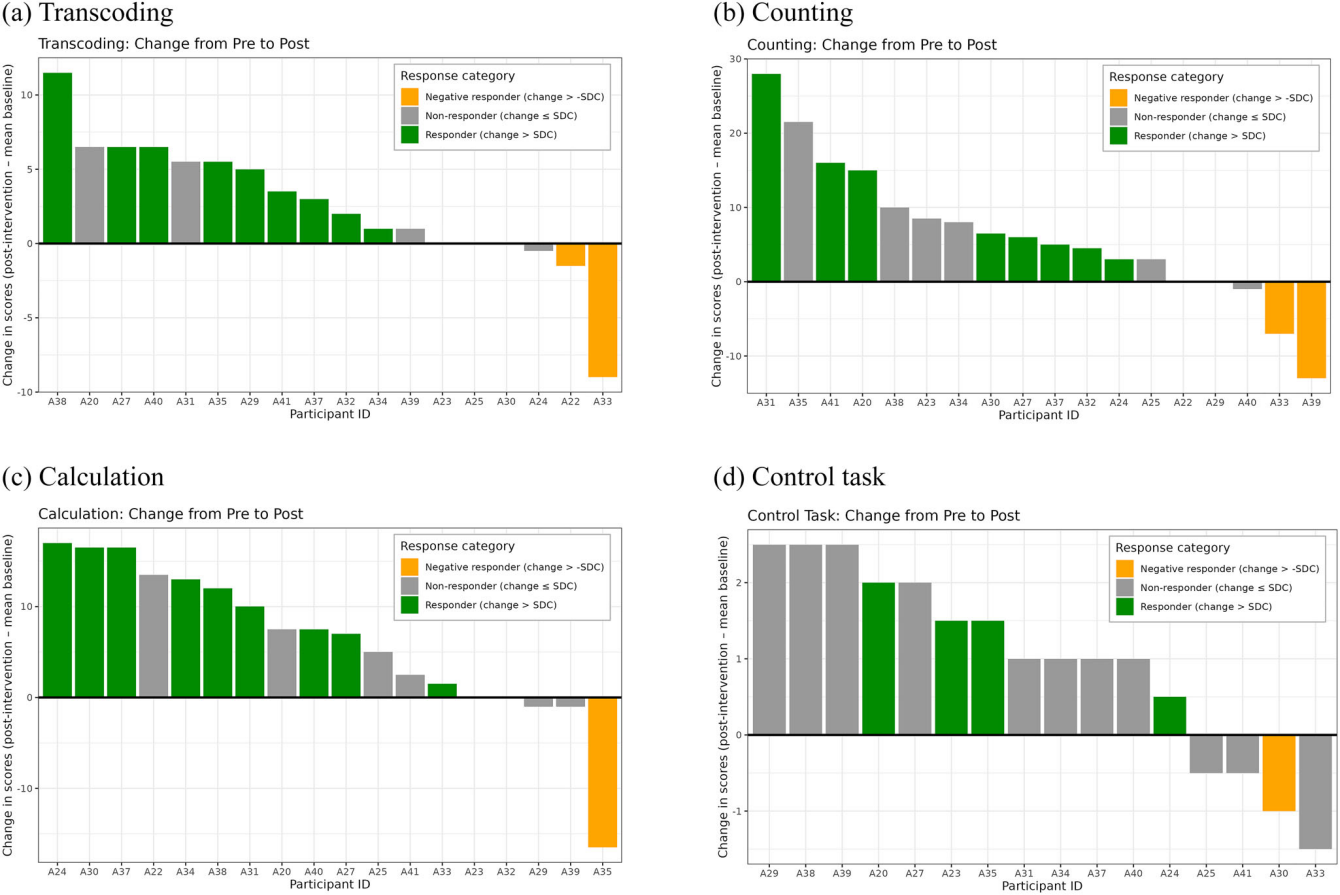
Plots depicting change in scores between baseline and immediately post‐intervention on transcoding, counting, calculation and control measures.

Table 3 shows the mean scores for the group and fixed effects results for transcoding, counting, calculation and the control task. The plots shown Figure 5 show change in individuals’ scores immediately post‐intervention on tests of transcoding, counting, calculation and the control task. They also highlight those identified as responders/non‐responders across the tasks (i.e. where improvement exceeded SDC).

**TABLE 3:** Results of mixed effects models for outcome measures.

Timepoint comparison with baseline 1	Mean (s.d.)	Estimate (*β*)	Std. error	df	t	*p*
Transcoding						
Intercept (baseline 1)	41.1 (18.2)	41.06	4.30	16.96	9.55	<0.001
Baseline 2	44 (17.8)	2.94	1.19	48.00	2.46	0.017
Post‐intervention	44.9 (17.2)	3.88	1.19	48.00	3.25	0.002
Follow‐up	45.3 (17.7)	4.24	1.19	48.00	3.55	<0.001
**Counting**						
Intercept (baseline 1)	41.3 (27.7)	46.80	5.93	18.27	7.90	<0.001
Baseline 2	41.6 (26.0)	.20	3.46	42.00	0.06	0.95
Post‐intervention	46.9 (27.0)	6.33	3.46	42.00	1.83	0.07
Follow‐up	48.3 (28.1)	7.93	3.46	42.00	2.29	0.03
Calculation						
Intercept (baseline 1)	37.1 (16.2)	37.12	4.39	19.08	8.46	<0.001
Baseline 2	41.1 (18.2)	3.94	2.10	48.00	1.88	0.06
Post‐intervention	46.6 (20.0)	9.47	2.10	48.00	4.52	<0.001
Follow‐up	43.4 (17.7)	6.24	2.10	48.00	2.98	0.004
Control task						
Intercept (baseline 1)	13.9 (2.81)	13.93	0.57	34.65	24.54	<0.001
Baseline 2	15.3 (2.19)	1.33	0.59	42.00	2.25	0.03
Post‐intervention	15.4 (2.23)	1.47	0.59	42.00	2.47	0.02
Follow‐up	15.7 (1.29)	1.73	0.59	42.00	2.92	0.006

#### 3.2.1 Transcoding

The linear mixed‐effects model showed a significant main effect of time, *F*(3, 48) = 5.19, *p* = 0.003, *ŋ*_p_^2^ = 0.24, with scores at post‐intervention and follow‐up significantly higher than at Baseline 1. However, improvements were also observed between the baselines. The residual variance was 12.12 (s.d. = 3.48), and the random intercept variance for participants was high (302.13, s.d. = 7.38) indicating substantial variability between participants and consistent with a low marginal *R* ^2^ (0.009) and high conditional *R* ^2^ (0.962). Nine participants were identified as responders with respect to transcoding (i.e. with an improvement in performance between baseline and post‐intervention greater than the SDC)—see Figure 5a. Some participants exhibited declines in performance on tasks following the intervention; however, several of these changes did not exceed the SDC threshold and therefore cannot be interpreted as meaningful. Two participants showed meaningful declines (i.e. exceeding SDC) in transcoding (indicated by orange bars in Figure 5a).

#### 3.2.2 Counting

The overall effect of time approached significance, *F*(3, 48) = 2.72, *p* = 0.054, *ŋ*_p_^2^ = 0.15. Fixed‐effects estimates showed no significant difference between scores at Baselines 1 and 2. Relative to the baseline, a marginal improvement in scores was observed post‐intervention, with a significant increase after the maintenance period (Table 3). The residual variance was 89.79 (s.d. = 9.48), and the random intercept variance for participants was estimated at 437.01 (s.d. = 20.91), again, consistent with a low marginal *R* ^2^ (0.013) and high conditional *R* ^2^ (0.892). In addition, individual analysis suggests that eight participants show evidence of responding to the intervention, with two participants exhibiting a decline in scores (Figure 5b). The two participants with the lowest pre‐intervention numeracy scores (A22, A40) performed at floor in this task.

#### 3.2.3 Calculation

Results of the model indicate a significant main effect of time on calculation performance, *F*(3, 48) = 7.23, *p* < 0.001, *ŋ*_p_^2^ = 0.31, Relative to Baseline 1, there was a marginally significant improvement in performance at Baseline 2, but significant increases both at post‐Intervention and follow‐up (Table 3). The residual variance was 37.32 (s.d. = 6.11). Again, the random intercept variance for participants was high, estimated at 290.16 (s.d. = 17.03), and the conditional *R* ^2^ was high (0.890) with a low marginal *R* ^2^ (0.036). Nine individuals showed evidence of an improvement in performance between baseline and post‐intervention greater than their individual SDC, with one individual (A35) showing a decline in performance greater than their SDC (Figure 5c).

#### 3.2.4 Functional Numeracy

There was no statistically significant difference between Baseline 2 (Mean = 14.8, s.d. = 3.05) and post‐intervention (Mean = 15.2, s.d. = 3.73) for this task (*t*(14) = 1.07, *p* = 0.30, Cohen's *d* = 0.28).

#### 3.2.5 Control Task

Results of the model indicate a significant effect of time for the control scores, *F*(3, 42) = 3.40, *p* = 0.026, *ŋ*_p_^2^ = 0.20. There were small but significant improvements in performance at all timepoints in comparison with Baseline 1. The residual variance was 2.64 (s.d. = 1.63), and the variance of random intercepts for participants was 2.19 (s.d. = 1.48) indicating moderate variability across participants, also reflected in the relatively modest conditional *R* ^2^ (0.500) and marginal *R* ^2^ (0.086). Despite the group level effect, and the fact that many participants show a numerical improvement in their score after intervention (Figure 5d), this change exceeded SDC for only four individuals.

Given the relatively small number of participants in the study and the possibility of type II errors across our main analyses, we conducted a post‐hoc power analysis using the simr package in R (Green and MacLeod 2016) and based on 1000 model simulations (*α* = 0.05). This indicated that power was high for Transcoding and Calculation (>90%), moderate for the Control task (82.5%), but low for Counting (64%), suggesting that the study was underpowered to detect group changes in the Counting task.

### 3.3 Gameplay Data

A summary of key variables across the group is presented in Appendix D. Data allowed examination of individuals’ adherence to the intervention, by observing the total amount of time spent playing the game (“Dosage”). This indicates that one person fell just short of the requested amount of 3 h and 45 min; all other participants exceeded what was requested, with a median dosage of 6 hours and 45 min. Some participants far exceeded the minimum dosage required with three playing for over 10 hours.

Five individuals (all with moderate to good pre‐intervention abilities) completed all 140 levels, but variation was substantial, with others reaching levels 50 to 130. Repeated levels were rare, with most participants easily able to progress (mean scores per level clustered above 90%), and sequence proportion showed uniformly high values. Time per tile (rate of play) fell generally between 1 and 2.5 seconds, but one outlier (A29) took 4.4 seconds per tile. The distribution of mean errors per level was between 4 and 7 for the great majority of participants, while one outlier (A29) reached 20.26. In order to explore the relationship between gameplay variables and overall response to therapy we visually examined boxplots to compare degree of overlap in the distribution of each gameplay variable between overall responders and non‐responders to therapy (see Appendix D). Despite the variations described above, no clear relationships were found between gameplay and outcomes.

### 3.4 Participant Feedback

Feedback on the intervention was generally positive and no adverse events were reported. Ten participants reported that they enjoyed SWAN for the whole treatment period, six people enjoyed it sometimes. Only one participant provided entirely negative feedback, describing the game as “Boring boring boring”. Some participants commented that the game was too easy (7), some that it became repetitive (6), although for one person the game became more challenging (“In the beginning it seemed too easy, but it became more challenging and interesting. I wanted to continue to play”). Participants reported greater confidence in their numeracy ability post‐intervention, for example,

“I think I'm quite good so it's relatively easy for me but it made me more confident”

“I feel more confident and can get to numbers faster. I can say 11–20 more easily now.”

We also asked participants what they would change about SWAN. In addition to the development of further gameplay components (e.g. leaderboard, more rewards), they made suggestions which would enable the intervention to provide greater support: the provision of more within‐game feedback on performance (10), including the spoken production of number names (10) and work with triple digit numbers (6).

## 4 Discussion

This study provides preliminary evidence that a novel game‐based intervention can be delivered to individuals with aphasia and is acceptable to them, and that it may be effective for re‐learning foundational numeracy skills.

### 4.1 Feasibility of the Intervention

The recruitment and retention rates indicate that it was feasible to recruit people with aphasia and to retain them in the study. 86% of the people who approached us about the research joined the study. The online nature of the study was not originally planned but meant it was possible to recruit people beyond the immediate study location who would not otherwise have been able to take part.

There was a high completion rate (89%), which may reflect the fact that individuals were experiencing difficulties that had not been addressed and so were highly motivated. In addition, the fact that the assessments and intervention took place at home and could be scheduled flexibly is likely to have facilitated recruitment and retention. Of the two individuals who did not complete the whole study, neither dropped out because of dissatisfaction: one withdrew from the study after baseline assessment because of other commitments, the other (A35) completed the intervention but was not able to take part in the follow‐up assessment as mentioned above.

Challenges presented by the Covid‐19 pandemic were met by developing online assessments. In terms of technical feasibility, therefore, we found that it was possible to conduct the assessments in this way, and despite previous reports (Hill et al. 2009), including individuals with severe aphasia. Careful planning of sessions and anticipation of potential areas of difficulty were essential: any difficulties manipulating the computer mouse were identified and addressed in an initial session—although of course it is still possible that not all errors resulting from mouse inaccuracy were avoided. All individuals were able to carry out the intervention independently at home: after the training session no one reported difficulties with use of the tablet or playing the game.

Gameplay data confirmed high levels of adherence with the intervention: 17/18 participants played for longer than required—in some cases much longer. This points to the success of gamification in facilitating repeated practice of the target behaviours. High adherence provides evidence of intervention acceptability, as does the feedback gathered from the participants, with all but one person reporting that they enjoyed the game.

A limitation to the current study relates to participant age: the average age of individuals in this study was somewhat younger than the typical age of stroke onset. Caution is therefore needed in considering how the findings may extend to older adults, who may differ in cognitive profile and familiarity with technology. The fact that post‐intervention gains were observed across the range of ages represented is encouraging, but future work should therefore extend the evaluation of the feasibility and acceptability of SWAN to older individuals with aphasia to determine whether similar engagement and gains can be achieved. Further evaluation of SWAN would also usefully include individuals with a greater range of aphasia profiles. The participant group here did not include individuals with a typical Wernicke's aphasia profile. This was not an intentional exclusion, but the absence of this group limits conclusions about the feasibility of SWAN for people with severe comprehension deficits.

### 4.2 Pre‐Intervention Numeracy Abilities

Data collected at baseline revealed marked variations in the presentation of numeracy deficits across individuals, aligning with previous research in this area (e.g. Marangolo et al. 2004, Marangolo et al. 2005).

Correlational findings indicated a mapping between patterns of numeracy deficit and language profiles, and those with relatively good numeracy abilities tended to have better language skills than those in the other groups. Previous research indicates, however, that the relationship between numeracy and language skills is not straightforward (e.g. Varley et al. 2005), and indeed in this study exceptions were noted. For example, participant A32 presented with relatively mild anomic aphasia but exhibited severe difficulties across all numerical tasks.

Baseline assessments also revealed variation within participants in terms of their performance, echoing similar variability found by Lemanissier and colleagues (2023). This may suggest that at least some of the behaviours observed are indicative of retrieval problems, rather than problems with storage of numerical information. Further research with a higher number of assessment points would allow greater examination of within‐participant variation, as well as provide a strong base on which to explore the replicability of the outcomes described here.

### 4.3 Preliminary Efficacy

The effects of SWAN on performance at the group level were evaluated using linear mixed effects models. Given the heterogeneity across our sample with respect to baseline abilities, we followed up these analyses by identifying individual participants whose improvement in performance post‐intervention exceeded the SDC. We found evidence of improvement in both proximal and distal assessments and in individuals with impairments across modalities.

#### 4.3.1 Proximal Measures

A significant improvement in transcoding following the SWAN intervention was observed, with scores at both post‐intervention points significantly higher than Baseline 1. However, performance also improved between the two pre‐intervention baselines, suggesting significant changes at the group level reflected general improvement over the course of the study. The high random intercept variance highlights substantial variability between participants. Post‐intervention improvements greater than SDC were observed for eight of the 18 participants (excluding the individual who also showed improvement in the control task). Previously published intervention studies have shown that transcoding difficulties can be ameliorated with intervention (e.g. Lemanissier et al. 2023): our findings support and extend these suggesting that improvements can be achieved without a complex, individualised intervention programme.

In other cases, experience of the game failed to trigger improvements in transcoding. Our assumption was that sequence knowledge was not modality‐specific, which meant that we did not test sequence knowledge directly using a task that resembled the SWAN game. Inclusion of a task requiring participants to identify a numerical sequence within a larger array of numbers might have enabled us to detect specific improvements that weren't transferred to the outcome measures we used (cf. Braley et al. 2021). In addition, adaptations to the game, including voice recognition, for example, would allow investigation of whether both output and input processing must be targeted if individuals are to make maximal gains.

The counting measure revealed no significant change between the two baseline points, indicating stable performance prior to intervention. A marginal improvement was observed post‐intervention, with significant gains evident after the maintenance period. This delayed effect suggests that counting skills may require a longer period of consolidation before measurable improvements emerge. There was substantial variability across participants, with six individuals classified as responders. The count sequence was directly trained by SWAN but gameplay did not require individuals to say aloud number names as was required in the outcome measure. It is possible that individuals elected themselves to repeat the items as they heard them in sequence or, where production was not hindered by apraxic difficulties, multiple inputs of items were sufficient to aid the re‐establishment of relevant sequences. Research exploring users learning through computer games routinely involves in‐person observation of their gameplay in a professional UX laboratory (e.g. Iacovides et al. 2015), and this may provide useful information about how learning relates to player involvement with SWAN.

It was predicted that the multiple opportunities to practise problematic numbers and sequences provided by SWAN would aid Hebbian learning. Note also that practice was marked by being largely free from error. It is important to note that the gameboards on which the tiles are presented in the SWAN game are arranged to constrain the decision process: every consecutive choice is made within the limited set of adjacent tiles. Therefore, while learning is not entirely error‐free, the game is designed to minimise error and maximise opportunity to select the correct number in sequence. Where beneficial, SWAN may therefore be viewed as acting as a scaffold for individuals, supporting existing associations or restoring those that are degraded.

Five individuals registered no change post‐intervention in the measures evaluating foundational number skills. This group includes three participants with relatively good abilities (A23, A25, A39), which suggests a possible ceiling effect for the best‐performing participants and that the scope for measurable improvement was extremely limited in these individuals. The participant with the most severe difficulties (A22) also showed no improvement after the intervention. Both A22 and A39 exhibited a decrease in scores which exceeded the SDC. For A39 this may be consistent with ceiling effects and regression to the mean. For A22 the drop in performance may reflect instability around floor‐level performance rather than true deterioration. Participant A33, with moderate baseline abilities, also showed a notable decline in both transcoding and counting post‐intervention. Examination of his gameplay showed that he reached only level 50 in the game, and although his total playtime exceeded the minimum, it was low relative to the rest of the participant group. This perhaps suggests he did not find the intervention helpful or that there were possible external factors affecting his ability to engage with SWAN. For these individuals then, SWAN may not be operating at the level required. The required dose was relatively low and even though most participants exceeded this, it falls below that reported by other intervention studies in this area of at least 20 hours (e.g. Ablinger et al. 2006). The design of the game meant participants had to progress in a linear fashion: the requirement to repeat a level was based on the success of the player to complete sequences successfully which they usually did. The game design did not anticipate the fact that for many people, problems captured in the outcome measures did not translate to multiple errors in SWAN. The effectiveness of SWAN may be improved with the inclusion of algorithms allowing players to progress through the game according to their abilities.

#### 4.3.2 Distal Measures

Calculation scores increased significantly at both post‐Intervention and follow‐up compared to Baseline 1, with marginal gains observed between the two pre‐intervention baselines. These results suggest that while calculation skills at the group level were relatively stable pre‐intervention, SWAN was effective in producing both immediate and sustained improvement even in the context of diverse baseline abilities. As with the proximal measures, we examined improvements at an individual level and observed gains greater than the SDC in eight participants. These captured individuals’ ability to complete more problems within the time limit after the intervention than before. SWAN does not directly address calculation skills, though a motivation for the inclusion of these in the outcome measures was evidence from developmental literature that transcoding and number sequence knowledge underly those later skills (e.g. Habermann et al. 2020). Addition and subtraction may be supported by later levels of the game which provide practice in “skip‐counting” in threes and fours as well as twos and fives. Models of numerical processing and arithmetic suggest two pathways for solving arithmetic problems in adults: a direct retrieval route may be used for over‐learned calculations (e.g. 3 + 4; 5–2); if the answer cannot be retrieved, then an alternative calculation procedure must be employed (e.g. McCloskey et al. 1986). Where retrieval is disrupted, as in cases of neurological damage, the individual may resort to strategies learned in childhood such as “counting on” to generate the solution (e.g. Warrington 1982). Use of such strategies is more time‐consuming so they may only be marked in speeded tasks such as the one used in this study. If practice with SWAN facilitates better access to numerals and the count sequence, it may subsequently result in greater fluency where the procedure pathway is required for arithmetic.

Importantly, there were no significant gains on functional numeracy, although several participants provided anecdotal reports of improvements in confidence and (to a lesser extent) use of numbers after the SWAN intervention. Because these data were collected anonymously to encourage candid feedback about the intervention, we were unable to determine whether the individuals offering these reports were classified as responders. Further work is therefore needed to explore whether greater practice on SWAN facilitates functional improvement or if targeted practice is required which places the abilities being trained within daily life contexts.

While the results of the current study are encouraging, they warrant further exploration before firm conclusions can be drawn about SWAN's effectiveness and the individuals for whom it may be most appropriate, particularly given the modest sample size and the associated impact on statistical power. One limitation of this study is that the outcome measures were custom‐designed. This includes the non‐numerical control task which was developed by drawing on assessment materials commonly used in the United States and was designed to be administered online. The task showed good internal consistency, but the gradual improvement across all testing points suggests that test‐retest reliability could be improved, although changes were modest compared to the targeted numeracy tasks.

Published numerical processing and calculation batteries are available (e.g. Delazer et al. 2003), but the full psychometric properties of these are yet to established. We elected to design our own measures for this study which explicitly probed areas of difficulty which have been reported in the literature (e.g. teens, decade numbers). The tests showed good internal consistency and strong positive correlations with the functional assessment, but further work is needed to establish measures required to evaluate interventions which are demonstrably psychometrically sound. This work could also incorporate the Numerical Abilities for Daily Living (NADL; Semenza et al. 2014) test as an additional baseline measure to provide a more comprehensive picture of each individual's functional numeracy abilities.

Further exploration of SWAN's effectiveness at an individual level is also required, which would allow closer examination of gameplay. While there were no relationships between improvements on outcome measures and gameplay variables at the group level, more detailed analysis possible at the individual level might reveal relationships between specific areas of difficulty and gameplay behaviour.

Overall, therefore, the feasibility data reported here provide impetus for further exploration of the effectiveness of SWAN. The study has confirmed the importance of unmet needs for numeracy support in aphasia and provided indications of SWAN's potential to address some of these challenges. Further data needs to be collected to establish whether these findings can be replicated or indeed surpassed when the intervention is delivered in less challenging conditions and where individual performance and gameplay are more closely monitored.

## Funding

This work was supported by a research grant awarded to the authors by the Nuffield Foundation (FR‐21731).

## Ethics Statement

Ethical approval was granted for this study by the UCL Research Ethics Committee, ref. 0430/007.

## Consent

Informed consent was obtained from all participants prior to beginning the study.

## Conflicts of Interest

The authors declare no conflicts of interest.

## Supporting information




**Supporting File 1**: jlcd70256‐supp‐0001‐SuppMat.docx
Appendix A. Stimuli in unpublished numerical tasks

**Supporting File 2**: jlcd70256‐supp‐0002‐SuppMat.docx
Appendix B. Scoring rules for the Counting assessment

**Supporting File 3**: jlcd70256‐supp‐0003‐SuppMat.pdf

**Supporting File 4**: jlcd70256‐supp‐0004‐SuppMat.docx
Appendix D. Key SWAN Gameplay Variables: definitions

**Supporting File 5**: jlcd70256‐supp‐0005‐SuppMat.docx
Appendix E. Further details on the nature of numerical impairment in SWAN participants

## Data Availability

The data that support the findings of this study are openly available on the Open Science Framework at https://osf.io/ag27j/.

## Endnotes

1 Because of the number of sessions participants were required to attend before the intervention started, the WAB was administered in the post‐intervention phase of the study.
