# Supplementary material for: Evaluation of the SWAN Game‐Based Approach to Re‐Building Numeracy Skills in Aphasia: Feasibility and Preliminary Findings
Source: Int J Lang Commun Disord. 2026 Apr 26;61:e70256. doi: 10.1111/1460-6984.70256 (PMC13111786; doi:10.1111/1460-6984.70256)
Supplement: Supplementary file 1 — Supporting File 1: jlcd70256‐supp‐0001‐SuppMat.docx Appendix A. Stimuli in unpublished numerical tasks [file JLCD-61-0-s001.docx]

**Appendix A. Stimuli in unpublished numerical tasks**

| **Task** | **Instructions** | **Items** |
| --- | --- | --- |
| **Counting** |  |  |
| Forwards | Count from 3 until I say stop. | 3-4-5-6-7  10-11-12-13-14  17-18-19-20-21  26-27-28-29-30  59-60-61-62-63  98-99-100-101-102  145-146-147-148-149  327-328-329-330-331 |
| Backwards | Now I am going to ask you to count backwards. For example starting at 3 and counting back, 3, 2, 1. Ready to continue?  Count back from 9 until I say stop. | 9-8-7-6-5  14-13-12-11-10  22-21-20-19-18  41-40-39-38-37  60-59-58-57-56  82-81-80-79-78  127-126-125-124-123  453-452-451-450-449 |
| **Transcoding** |  |  |
| Number writing | I will say a number, and you should write it down. | 5, 9, 13, 14, 15, 18, 19, 36, 40, 60, 70, 71, 134, 295, 380, 508, 620, 746, 807, 905 |
| Number identification | You will see four numbers on the screen Click on the play button to listen to a number. Click on the number that you just heard. If you are not sure, you can listen to the number again. | 13, 15, 16, 19, 28, 37, 41, 70, 80, 85, 168, 206, 356, 435, 573, 614, 670, 784, 807, 940 |
| Number reading | You will see a number on the screen. Tell me which number it is. | 7, 9, 13, 16, 17, 18, 19, 37, 40, 50, 52, 74, 130, 201, 384, 578, 614, 706, 815, 960 |
| **Calculation** |  |  |
| Simple addition | In the first test you will do additions. On the screen you will see an equation. Type your answer in the box next to it. This test is timed. You will have 60 seconds. Try to answer as many as possible. | 2+1, 1+4, 1+2, 4+1, 3+5, 2+3, 1+3, 4+2, 3+1, 2+7, 4+3, 1+5,  6+2, 2+8, 3+6, 5+4, 2+5, 5+2,  7+2, 4+5, 1+7, 3+3, 4+4, 2+6, 8+1, 5+5, 6+3, 2+2, 5+3, 8+2, 5+4, 2+5, 5+2, 7+2, 4+5, 1+7, 3+3, 4+4, 2+1, 1+4, 1+2, 4+1, 3+5, 2+3, 1+3, 4+2, 3+1, 2+7, 4+3, 1+5, 6+2, 2+8, 3+6, 2+6, 8+1, 5+5, 6+3, 2+2, 5+3, 8+2 |
| Addition with carry | The next test is about additions, but this time with bigger numbers. On the screen you will see an equation. Type your answer in the box next to it. You have 60 seconds. Try to complete as many calculations as you can. | 9+2, 8+4, 5+7, 7+4, 6+5, 9+7, 5+9, 6+8, 6+6, 5+8, 7+8, 6+5, 7+7, 8+9, 4+6, 3+8, 2+9, 9+8,  7+6, 8+5, 8+7, 6+9, 3+9, 6+7, 9+9, 7+9, 9+3, 6+7, 8+4, 9+7, 6+9, 3+8, 7+6, 9+4, 5+8, 9+9, 8+4, 6+9, 7+7, 5+9, 6+5, 2+9, 8+5, 7+8, 9+3, 5+7, 6+9, 9+3, 6+5, 7+8, 6+6, 8+4, 7+6, 5+9, 9+9, 6+8, 4+9, 6+7, 5+6, 9+2 |
| Simple subtraction | The next test is about subtraction. Again, you have 60 seconds to complete as many equations as you can.  Try to answer as many as possible. | 2-1, 4-1, 4-2, 7-3, 6-1, 5-2, 6-4, 3-1, 6-5, 7-4, 3-2, 7-5, 8-3, 7-1, 9-3, 8-5, 9-5, 5-4, 6-2, 9-3, 9-2,  7-2, 6-3, 9-6, 6-5, 9-4, 5-3, 8-6, 5-1, 9-8, 8-5, 9-5, 5-4, 6-2, 9-3, 9-2, 7-2, 6-3, 3-1, 6-5, 7-4, 3-2, 7-5, 8-3, 7-1, 9-6, 6-5, 9-4, 5-3, 8-6, 5-1, 9-8, 2-1, 4-1, 4-2, 7-3, 6-1, 5-2, 6-4, 3-1 |
| Subtraction with carry | This is the last test. This one is about subtraction, this time with bigger numbers. Again, you have 60 seconds to complete as many as you can. | 14-5, 15-9, 11-2, 13-5, 11-6,  15-6, 16-7, 17-8, 11-5, 17-8,  18-9, 11-4, 11-8, 12-7, 12-9,  14-8, 13-5, 18-9, 16-8, 14-6  12-4, 13-9, 16-7, 11-7, 16-9,  14-7, 15-8, 18-9, 11-9, 17-9,  15-8, 11-7, 18-9, 11-4, 11-9,  14-5, 17-8, 13-5, 16-9, 15-6,  12-4, 13-8, 18-9, 11-5, 13-9,  12-6, 16-7, 13-4, 17-8, 15-9,  11-6, 18-9, 13-5, 12-9, 16-8,  11-2, 17-9, 13-6, 15-8, 11-8 |

**2. Counting scoring procedure**
