# Supplementary material for: Evaluation of the SWAN Game‐Based Approach to Re‐Building Numeracy Skills in Aphasia: Feasibility and Preliminary Findings
Source: Int J Lang Commun Disord. 2026 Apr 26;61:e70256. doi: 10.1111/1460-6984.70256 (PMC13111786; doi:10.1111/1460-6984.70256)
Supplement: Supplementary file 2 — Supporting File 2: jlcd70256‐supp‐0002‐SuppMat.docx Appendix B. Scoring rules for the Counting assessment [file JLCD-61-0-s002.docx]

**Appendix B. Scoring rules for the Counting assessment**

1. Award 1 point for each number in the count sequence produced.
2. Maximum score = 80 (Count forwards = 40; Count backwards = 40)
3. Rater 1 is unblinded and Rater 2 is blind to participant ID and timepoint.

Repetition of stimuli:

- If the participant required 1 repetition of the starting number = score sequence normally
- If the participant required >1 repetition of the starting number = score sequence as 0

Starting a sequence:

- If the participant does not directly go into the sequence (e.g. 20, 20, 26, 27, 28 where the starting point of the sequence was 26) = score as 0 correct
- If the participant only repeats the starting number/prompt but did not continue the sequence = score as 0
- If the participant repeats the starting number and continued sequence (e.g. 59, 59, 60, 61 etc) = score as 0
- If the participant does not include the prompt but continued the sequence (e.g. prompt = “145”, they say “146, 147, 148, 149 etc” = include starting number in score (so max possible for sequence is still 5)

Self-correction:

- Stop scoring as soon as participant goes “off” sequence (e.g. 57, 58, 59, 61 eh no, 60, 61 = stop scoring as correct after 59 as it goes off sequence = 3 points)

Self-repetition:

- Where a participant repeats a number in sequence (e.g. 20- 19 -19 -18) = count first production (e.g. 19) as correct then stop scoring

Variants of the target number:

- If the participant numbers breaks down numbers into single or double digits (e.g. “3-2-0” instead of “320”; “1-6” instead of “16”; “3-28” instead of “328”) = score as incorrect
- If the participant produces apraxic-type speech errors (e.g. “mine” instead of “nine”) = score normally

Pausing:

- If the participant pauses mid-sequence or mid-number (including filled pause, e.g. “eh”) = score as correct

Prompts:

- If the participant needed to see the starting number written down to start sequence (to account for hearing/internet difficulties )= score normally
- If the participant writes down number / uses fingers to count / draws in the air = score as 0
