# Supplementary material for: Evaluation of the SWAN Game‐Based Approach to Re‐Building Numeracy Skills in Aphasia: Feasibility and Preliminary Findings
Source: Int J Lang Commun Disord. 2026 Apr 26;61:e70256. doi: 10.1111/1460-6984.70256 (PMC13111786; doi:10.1111/1460-6984.70256)
Supplement: Supplementary file 3 — Supporting File 3: jlcd70256‐supp‐0003‐SuppMat.pdf [file JLCD-61-0-s005.pdf]

TIDieR

The SWAN game-based approach to re-building numeracy skills in aphasia

Why:

Difficulty understanding and communicating numbers is a common problem for people with aphasia post stroke (e.g. De Luccia & Ortiz, 2014). Often, individuals can recognise numbers but are unable to name them aloud, or at least not without writing them or counting up from one. Despite the frequency of number difficulties, there are few available assessments or therapies targeting this area.

The SWAN therapy game addresses numeracy difficulties by enhancing foundational number language: the Arabic numeral sequence and the relationship between the spoken number name and Arabic numeral (i.e. transcoding). It is based on the stages of number acquisition identified in children (Fuson, 1988) and mirrors the counting up behaviour observed in some aphasic adults. The therapy exploits gaming technology to encourage intensive practice of skills distributed across multiple sessions, which has been found to be effective in other studies in aphasia (e.g. Dignam et al., 2016).

The aim of the study was to explore the effects of SWAN on the numeracy abilities of a group of people with aphasia, including objective number skills and the ability to carry out everyday tasks involving numbers.

References

De Luccia, G., & Ortiz, K. Z. (2014). Ability of aphasic individuals to perform numerical processing and calculation tasks. *Arquivos de Neuro-Psiquiatria*, *72*(3), 197–202. https://doi.org/10.1590/0004-282X20130250

Dignam, J.K., Rodriguez, A.D., & Copland, D.A. (2016). Evidence for intensive aphasia therapy: consideration of theories from neuroscience and cognitive psychology. PM & R : *The Journal of Injury, Function & Rehabilitation, 8*(3), 254–267. https://doi-org.libproxy.ucl.ac.uk/10.1016/j.pmrj.2015.06.010

Fuson, K.C. (1988). *Children's counting and concepts of number*. New York: Springer.

What (material):

The intervention focuses on the foundational skills of:
- **transcoding** between Arabic digits and spoken number names
- the **count sequence**, including forwards and backwards and skip counting (twos, threes, fours, fives and tens).

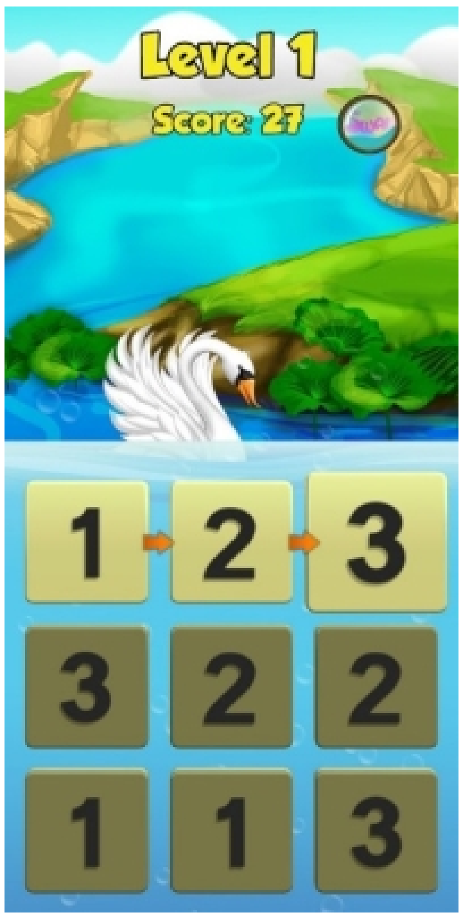

The core activity of the game is the identification the longest possible number sequence available by selecting adjacent tiles in consecutive order (see example screenshot from Level 1). Scores are awarded for each sequence completed, and are based on the length of the sequence relative to the maximum possible sequence. Every time the player touches an Arabic numeral on the screen they hear the corresponding spoken form. In order to provide support for players with more limited number knowledge, where a player is slow to start their turn 'help' arrows appear on screen indicating a possible sequence.

What (procedures):

Progression

Progression through the 140 levels of the game is constrained by an increase in difficulty across a set of variables, so that progression represents both the accumulation of number sequence knowledge but also the expansion of gameplay skills.

- Number range: ranges within 1-99 are included, following the stages of learning of the verbal number sequence as outlined by Fuson (1988), from the "unbreakable string" to entering the number sequence after 1, to bidirectional counting.
- Sequence length: higher levels provide the opportunity for longer sequences to be created.
- Game board size: progression of the SWAN levels includes board sizes from a 3x3 matrix of tiles up to 7x7 tiles.
- Additional gameplay elements: constraints on sequence construction include filler tiles ('water-lilies') and blank tiles which can stand for any number in a sequence. 'Shell' tiles bear a number but are distinguished from standard number tiles since they can be re-used, and don't disappear once the sequence within which they are used is cleared. Re-use of the shell increases the player's score. Deliberate use of shells indicates a higher level of skill.

Bonus games
Two bonus games are included at regular intervals as participants proceed through the levels of the core game. These offer additional practice in transcoding and count sequence (number-line) completion and are intended explicitly to reinforce these key aspects of number knowledge, as well as to enhance motivation for the player.

- Letter box game (transcoding practice): a letter is presented for "posting" in one of four numbered post boxes which appear on screen. As each letter is presented a spoken number is played (e.g. "seventeen") the player must match the letter to the correct numbered box. The number choices of the distractor post boxes are designed to represent phonological, semantic and syntactic similarities which have been found to pose particular challenges (e.g. 17-70; 17-71) (e.g. Messina et al., 2009).
- Number line game (count sequence practice): Bubbles containing Arabic numerals rise from the seabed. Players must 'grab' the number needed to complete a sequence displayed at the top of the screen and place it in the appropriate slot. Difficulty increases as the number of blanks in a sequence increases and the interval between bubbles appearing decreases.

A demonstration of SWAN can be viewed here: https://www.youtube.com/watch?v=1hy4k3IcaOQ

References

Fuson, K.C. (1988). *Children's counting and concepts of number*. New York: Springer.

Messina, G., Denes, G., & Basso, A. (2009). Words and number words transcoding: A retrospective study on 57 aphasic subjects. *Journal of Neurolinguistics*, *22*, 486–494. https://doi.org/10.1016/j.jneuroling.2009.04.001

Who provided: Training on the SWAN app – over Zoom – was provided to each participant individually by a member of the research team. This ensured that they could access the game on the tablet and that they understood the principles of the game before the intervention period began.

How (mode of delivery; individual or group): Intervention was delivered entirely via the tablet-based game. Where participants did not have their own Android tablet, one was sent to them via mail. All participants were sent an instruction booklet which they could refer to during the intervention period.

Where: Participants completed the SWAN intervention at home,

When and how much: Participants were asked to complete 15 minutes of SWAN playing time every weekday for the 3 weeks of the intervention period, totalling 3 hours 45 minutes of treatment.

Tailoring: As outlined above, the app provides multiple structured levels of difficulty. This is largely static in that all participants are required to complete fixed levels in order. Dynamic levelling is provided in that where the criterion for passing a level is not reached (based on their score relative to the maximum possible score) the player is required to repeat the level, thus allowing for further practice of areas which are particularly problematic for an individual.

Modification: No modifications to the SWAN intervention were made during the course of the evaluation study.

How well (planned): In-app gameplay data automatically collected by the SWAN app allowed us to examine individuals' adherence to the requested amount and intensity of the intervention, by observing the total amount of time spent playing the game.

How well (actual): Two participants (out of eighteen) fell just short of the requested amount of 3 hours and 45 minutes, but both registering over 3 hours of SWAN gameplay. All other participants exceeded what was requested, with a median dosage of 6 hours and 45 mins. In general this time was relatively evenly distributed across the intervention period. Some participants far exceeded the minimum dosage required with three participants recording over 10 hours of playing time.
