# Supplementary material for: Evaluation of the SWAN Game‐Based Approach to Re‐Building Numeracy Skills in Aphasia: Feasibility and Preliminary Findings
Source: Int J Lang Commun Disord. 2026 Apr 26;61:e70256. doi: 10.1111/1460-6984.70256 (PMC13111786; doi:10.1111/1460-6984.70256)

**Appendix D. Key SWAN Gameplay Variables: definitions**

| **Variable** | **Definition** |
| --- | --- |
| Maximum level reached | The number of the final level played (out of 140). This is an indicator of the extent of exposure to the game, and to the skill level of the player. Since a criterion score (70% of maximum possible score) is required for progression, this variable represents a composite based on the number, length and complexity of number sequences identified by the player within the constraints of gameplay rules, and within the three-week intervention period. |
| Number of repeated levels | The number of times a player failed to meet the criterion (70% of maximum possible score) for progression to the next level. The number of repeated levels is a general indicator of extent to which the game challenges the player’s gaming skill or number knowledge. Successful progression following repetition is an indicator of learning. |
| Mean sequence proportion | This measure is based on comparison of the length of each sequence entered with the maximum possible sequence length available on the current board. It provides an indicator of the player’s understanding and engagement with the aim of producing the longest possible sequence. |
| Mean score per level | The average percentage of the maximum possible score achieved by the player at each level. This is an indicator of the player’s ability to manage the specific task demands (gameplay skills and sequence knowledge) of the levels they played. |
| Mean time per tile | The average time taken (in seconds) for the player to select each tile in each sequence at each level played (excluding the time taken to select the first tile in each sequence). This is an indicator of rate of play and proficiency with the game. |
| Mean number of errors per level | A measure of accuracy of gameplay. Errors are defined as failures to observe gameplay rules. These may represent gameplay error per se (e.g. selection a non-adjacent tile), or else number sequence failure (selection of non-consecutive number). Errors may also be recorded as a result of technical failure. |
| Dosage | The total time spent engaging with the game. |
| Total therapeutic inputs | Following Harvey et al’s (2021) review of treatment dose in aphasia therapy, this measure captures the number of individual direct therapeutic inputs a player receives. It is measured by calculating the total number of tile presses registered by a player over the course of their engagement with SWAN. This variable is equivalent to the Cumulative Intervention Intensity value reported in Cordella & Kiran (2024). |
| *From Cordella & Kiran (2024)* |  |
| Dose | Number of therapeutic inputs per gameplay session |
| Frequency | Number of gameplay sessions per week |
| Session Duration | Number of minutes per gameplay session |

**Summary of key gameplay variables for the participants**

| **Pt** | **Maximum level reached** | **Number of repeated levels** | **Mean sequence proportion** | **Mean score** | **Mean time per tile (secs)** | **Mean number of errors per level** | **Dosage** | **Total therapeutic inputs** | **Dose** | **Frequency** | **Session Duration** |
| --- | --- | --- | --- | --- | --- | --- | --- | --- | --- | --- | --- |
| A20 | 70 | 1 | 0.94 | 0.88 | 1.55 | 4 | 04:51:30 | 5,752 | 304.00 | 4.50 | 971.66 |
| A22 | 55 | 1 | 0.95 | 0.89 | 2.7 | 6.07 | 07:07:27 | 4,866 | 283.25 | 5.33 | 1602.91 |
| A23 | 140 | 0 | 0.97 | 0.95 | 1.22 | 6.66 | 06:37:34 | 11,873 | 781.50 | 4.67 | 1703.84 |
| A24 | 113 | 0 | 0.95 | 0.92 | 1.21 | 2.84 | 05:05:08 | 9,509 | 612.53 | 5.00 | 1220.54 |
| A25 | 77 | 11 | 0.90 | 0.81 | 1.32 | 15.1 | 06:54:49 | 9,184 | 320.84 | 6.25 | 995.57 |
| A27 | 140 | 0 | 0.95 | 0.93 | 1.92 | 4.26 | 09:55:15 | 11,586 | 915.83 | 5.22 | 2976.26 |
| A29 | 74 | 7 | 0.92 | 0.83 | 4.44 | 20.26 | 17:42:36 | 7,534 | 375.44 | 5.16 | 3984.74 |
| A30 | 140 | 1 | 0.95 | 0.94 | 1.66 | 3.12 | 08:47:33 | 11,408 | 421.96 | 7.03 | 1217.42 |
| A31 | 129 | 1 | 0.92 | 0.87 | 2.41 | 10.76 | 15:05:00 | 11,525 | 482.71 | 5.38 | 2585.73 |
| A32 | 71 | 0 | 0.97 | 0.96 | 2.97 | 8.59 | 08:35:41 | 6,204 | 224.96 | 6.76 | 1237.65 |
| A33 | 50 | 0 | 0.96 | 0.95 | 2.11 | 3.38 | 04:05:57 | 4,172 | 307.92 | 5.00 | 1135.13 |
| A34 | 140 | 0 | 0.96 | 0.94 | 1.45 | 2.42 | 06:33:32 | 11,370 | 787.93 | 5.38 | 1686.60 |
| A35 | 105 | 1 | 0.95 | 0.91 | 2.41 | 5.25 | 10:10:40 | 9,340 | 488.28 | 6.00 | 2035.58 |
| A37 | 70 | 4 | 0.92 | 0.88 | 1.02 | 5.44 | 03:38:56 | 6,306 | 658.33 | 2.90 | 1459.54 |
| A38 | 40 | 0 | 0.96 | 0.91 | 2.29 | 5.78 | 04:10:03 | 3,272 | 190.06 | 5.16 | 937.71 |
| A39 | 140 | 0 | 0.97 | 0.95 | 0.78 | 2.91 | 04:37:46 | 11,402 | 845.77 | 5.65 | 1282.00 |
| A40 | 71 | 0 | 0.97 | 0.94 | 2.87 | 4.52 | 08:37:40 | 5,883 | 264.86 | 7.00 | 1479.05 |
| A41 | 110 | 4 | 0.92 | 0.87 | 1.09 | 6.75 | 05:14:00 | 10,734 | 475.76 | 5.38 | 897.16 |

**Pt = Participant**

**Heatmap showing distribution of gameplay across intervention sessions**

*(depth of blue relates to amount of playing time; white blocks represent days with no gameplay)*


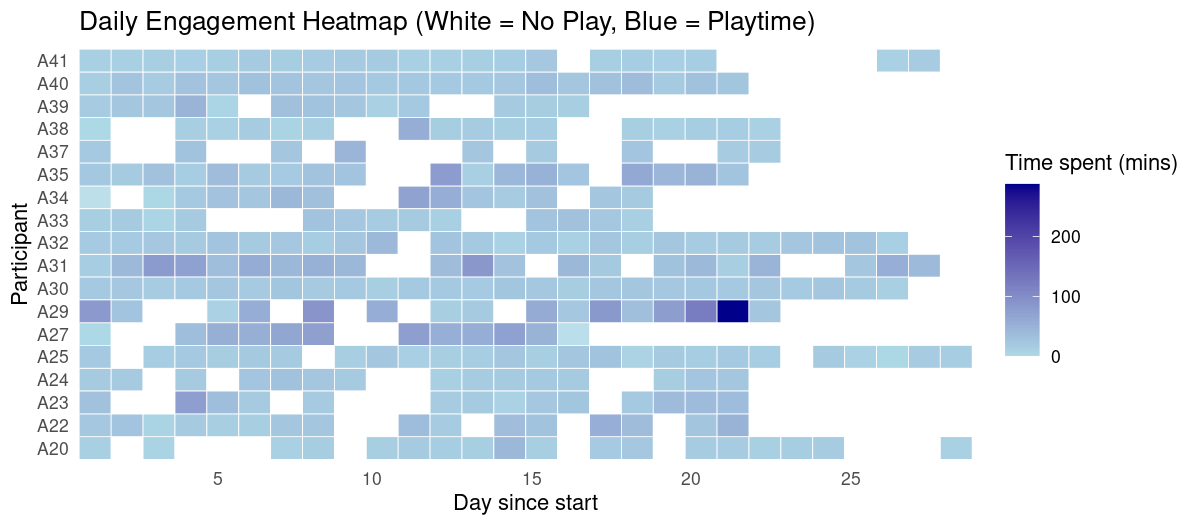


**Boxplots showing distribution of gameplay variables for individuals who responded to the intervention in at least one outcome variable versus those who did not respond to the intervention**


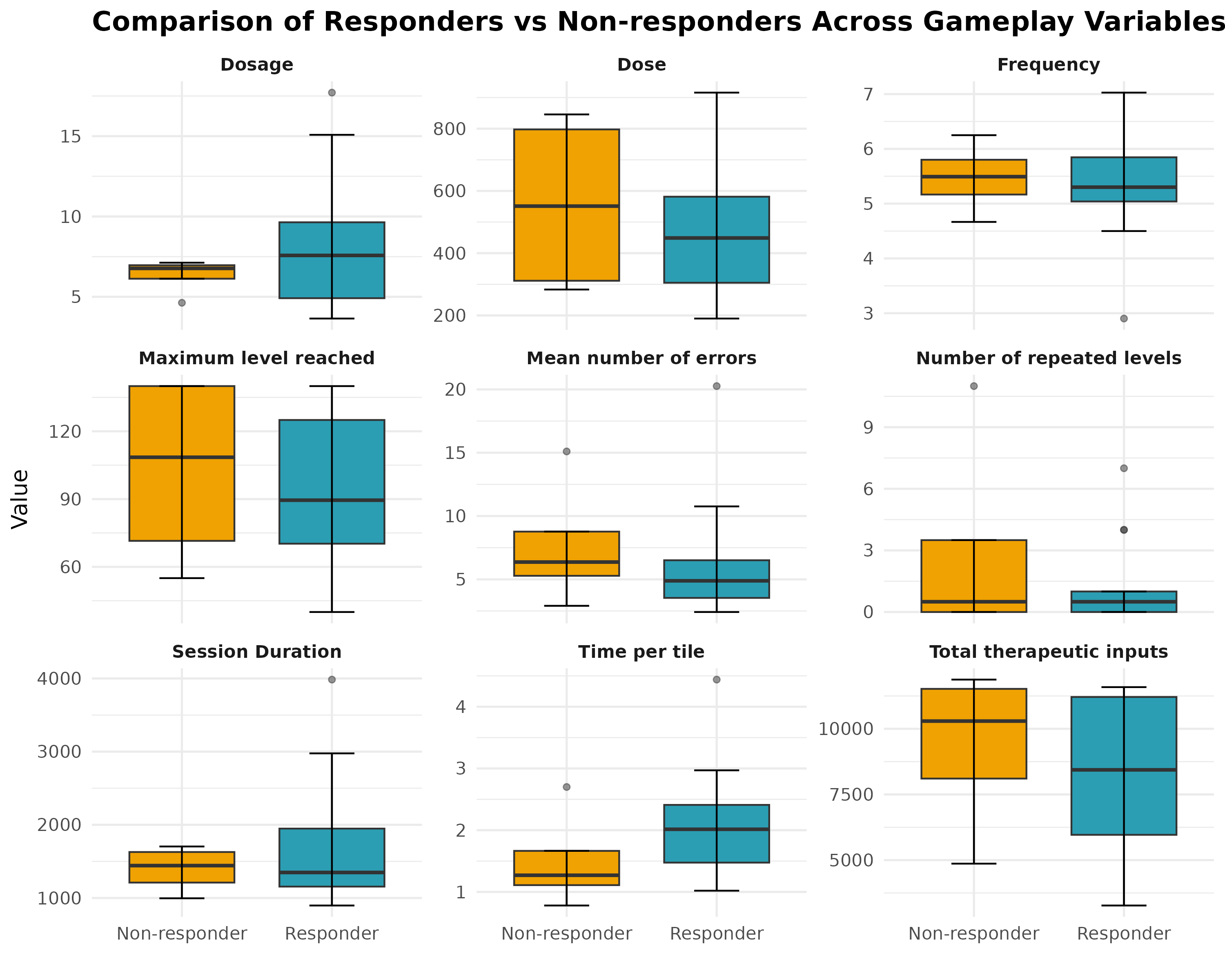

Supplement: Supplementary file 4 — Supporting File 4: jlcd70256‐supp‐0004‐SuppMat.docx Appendix D. Key SWAN Gameplay Variables: definitions [file JLCD-61-0-s003.docx]
