# Supplementary material for: Evaluation of the SWAN Game‐Based Approach to Re‐Building Numeracy Skills in Aphasia: Feasibility and Preliminary Findings
Source: Int J Lang Commun Disord. 2026 Apr 26;61:e70256. doi: 10.1111/1460-6984.70256 (PMC13111786; doi:10.1111/1460-6984.70256)
Supplement: Supplementary file 5 — Supporting File 5: jlcd70256‐supp‐0005‐SuppMat.docx Appendix E. Further details on the nature of numerical impairment in SWAN participants [file JLCD-61-0-s004.docx]

Appendix E. Further details on the nature of numerical impairment in SWAN participants

| **Pt** | **AQ** | **Aphasia type** | **Transcoding subtask^†^** | | | **Writing errors^‡^** | | | | |
| --- | --- | --- | --- | --- | --- | --- | --- | --- | --- | --- |
|  |  |  | ID | Reading | Writing | NR | Syntactic | Semantic | Phonological | Mixed |
| A20 | 92.9 | Anomic | 19.5 | 19.5 | 13.5 | 1 | 1 | 4 | 6 | 1 |
| A22 | 24.3 | Broca’s | 6.5 | 1 | 0 | 0 | 0 | 34 | 0 | 6 |
| A23 | 96.1 | Anomic | 20 | 20 | 20 | 0 | 0 | 0 | 0 | 0 |
| A24 | 78.3 | Anomic | 17.5 | 17 | 13 | 2 | 0 | 8 | 4 | 0 |
| A25 | 84.4 | Conduction | 17.5 | 20 | 16.5 | 5 | 0 | 2 | 0 | 0 |
| A27 | 71.1 | Conduction | 18.5 | 17.5 | 17.5 | 0 | 0 | 1 | 4 | 0 |
| A29 | 35.9 | Broca’s | 11 | 0.5 | 15.5 | 0 | 0 | 9 | 0 | 0 |
| A30 | 92.6 | Anomic | 20 | 20 | 18 | 2 | 0 | 1 | 1 | 0 |
| A31 | 69.7 | Conduction | 12 | 11 | 5.5 | 1 | 1 | 19 | 7 | 1 |
| A32 | 78.8 | Anomic | 7 | 5 | 4 | 22 | 0 | 9 | 0 | 1 |
| A33 | 84.4 | Anomic | 14.5 | 20 | 17.5 | 1 | 1 | 3 | 0 | 0 |
| A34 | 78.7 | Anomic | 19.5 | 18 | 18.5 | 0 | 0 | 2 | 1 | 0 |
| A35 | 45 | Broca’s | 16.5 | 4.5 | 8.5 | 0 | 1 | 19 | 2 | 1 |
| A37 | 96.4 | Anomic | 18 | 20 | 18 | 0 | 2 | 2 | 0 | 0 |
| A38 | 61.5 | Conduction | 13.5 | 12 | 7 | 9 | 2 | 11 | 4 | 0 |
| A39 | 93.2 | Anomic | 19 | 20 | 18 | 0 | 0 | 0 | 4 | 0 |
| A40 | 46.9 | Broca’s | 7.5 | 1.5 | 3.5 | 0 | 1 | 30 | 0 | 2 |
| A41 | 85.5 | Anomic | 17.5 | 17 | 18 | 0 | 3 | 1 | 0 | 0 |

Key: Pt = Participant; AQ = Aphasia Quotient from the Western Aphasia Battery; NR = No response

**^†^** Average scores across both baseline sessions

**^‡^** Total number of errors at Baseline 1 and 2

**Analysis of errors**

- No response = not attempted by the participant
- Syntactic errors = include expansion errors (the insertion of additional zeroes, e.g. “one hundred and three” 🡪 1003) and inversion errors (where the order of basic lexical elements inverted with respect to the Arabic notation, e.g. “five hundred and eight” 🡪 580)
- Semantic errors = the inaccurate choice of constituent digits in the stimulus, while preserving its overall order of magnitude (e.g. “fourteen” 🡪 18)
- Phonological errors = confusions between numbers whose names sound alike, e.g. “fourteen” 🡪 40)
